# Denture Impaction Causing an Upper Esophageal Diverticulum

**DOI:** 10.1155/2019/9621383

**Published:** 2019-07-16

**Authors:** Shireen Samargandy, Hani Marzouki, Talal Al-Khatib, Mazin Merdad

**Affiliations:** Department of Otolaryngology-Head and Neck Surgery, King Abdulaziz University, Jeddah, Saudi Arabia

## Abstract

**Background:**

Dentures are a common cause of inadvertent foreign body ingestion particularly in the elderly. Due to their radiolucent nature, they often present a diagnostic challenge to care providing physicians.

**Case Presentation:**

A 66-year-old female presented to our otolaryngology clinic with a 2-year history of dysphagia. Her physical examination was unremarkable. Computed tomography scan of the neck and barium swallow suggested Zenker diverticulum. She was planned for endoscopic diverticulotomy; however, during surgery, a foreign body was incidentally found and retrieved, which was a partial lower denture. The diverticulum resolved thereafter, and the patient's symptoms abated.

**Conclusion:**

The authors recommend evaluating the esophagus endoscopically first in cases of upper esophageal diverticular formation, even when planning an open repair approach, to rule out any concealed foreign bodies.

## 1. Introduction

Accidental foreign body (FB) ingestion is a common problem in children and certain elderly populations. Among edentulous populations with compromised mental status or neurological disabilities, dentures are a frequent cause of FB impaction in the esophagus. Dentures are notoriously challenging to diagnose due to their radiolucent nature. Although the use of radiopaque substances in denture manufacturing has been recommended, such materials have yet to be successfully used without compromising denture quality. Hence, there is often a delay in the diagnosis and management of patients with impacted dentures, predisposing them to higher risks of complications such as esophageal perforation, diverticulum or fistula formation, or obstruction of the bowel. A rise in the rate of complications from 3.2% to 23.5% was reported when comparing patients managed after 24 hours with ones managed after 48 hours [1]. There are several described cases of denture impaction in the aerodigestive tracts in literature; however, only a small number of cases presented with diverticular formation. Here we present a special case of prolonged esophageal denture impaction that was diagnosed as Zenker diverticulum and later discovered to be a denture embedded in the patient's upper esophagus.

## 2. Case Presentation

A 66-year-old female presented to the otolaryngology clinic at our institution with a 2-year history of dysphagia to solids and liquids that was unprogressive in course. Her complaint was associated with mild odynophagia and unmeasured weight loss, as well as occasional coughing, aspirations, and regurgitation. Her past medical and surgical history was unremarkable.

On examination, the patient looked well but slightly underweight. She had no issues in phonation or respiration. There was no neck tenderness or swelling. Her oral exam was unremarkable except for being partially edentulous and having a missing single lower incisor tooth. Flexible laryngoscopy was performed in clinic and was normal.

Prior to her presentation at our institution, the patient had a computed tomography (CT) scan of the neck done in another hospital which showed a well-defined air-filled space at the level of the cricopharyngeus (CP) muscle measuring 13x19x37 millimeters. The report suggested Zenker diverticulum. She was referred to our tertiary care center for surgical management.

**Figure 1:**
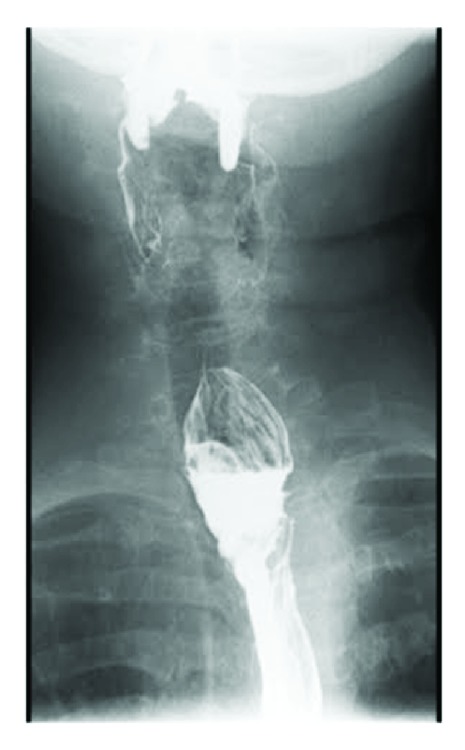
Barium swallow showing a left paramedian diverticulum with mild indentation on the trachea.

**Figure 2:**
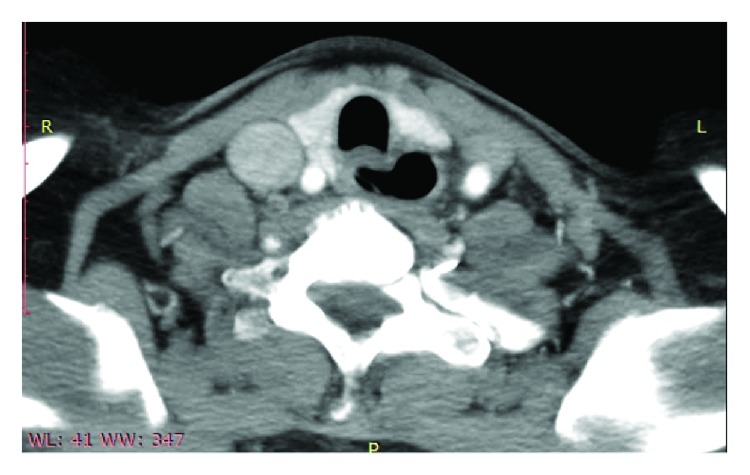
Axial CT scan image of the neck with contrast confirming the diverticulum arising from the midline that is directed towards the left side.

On admission, the patient's vital signs and basic laboratory blood investigations were within normal limits. Her chest X-ray was unremarkable. A barium swallow revealed a left paramedian diverticulum located at the thoracic inlet (Figure 1). The previous CT was reviewed, and the radiologist confirmed the presence of a cricopharyngeal diverticulum arising from the midline and directed towards the left side consistent with Zenker diverticulum (Figure 2).

Considering the patient's clinical scenario and imaging studies, the patient was offered definitive surgery in the form of endoscopic diverticulotomy, with the possibility of converting to an open approach if endoscopic exposure was inadequate.

**Figure 3:**
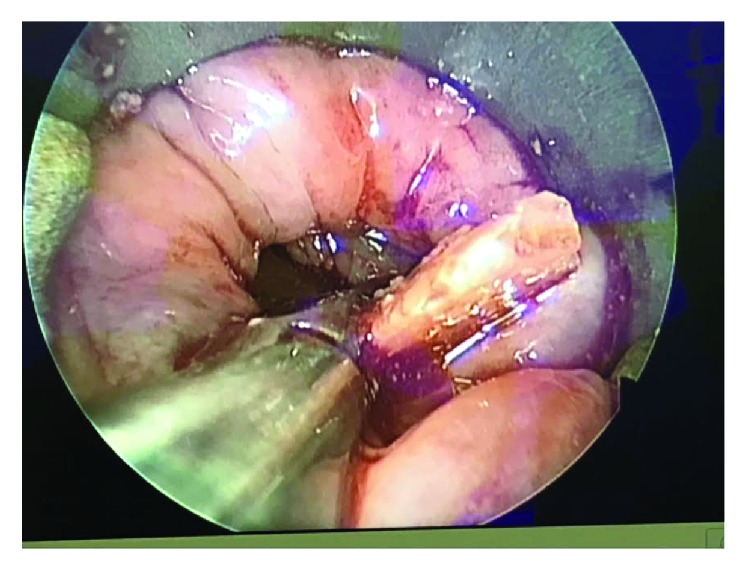
Endoscopic image of the foreign body retrieval.

**Figure 4:**
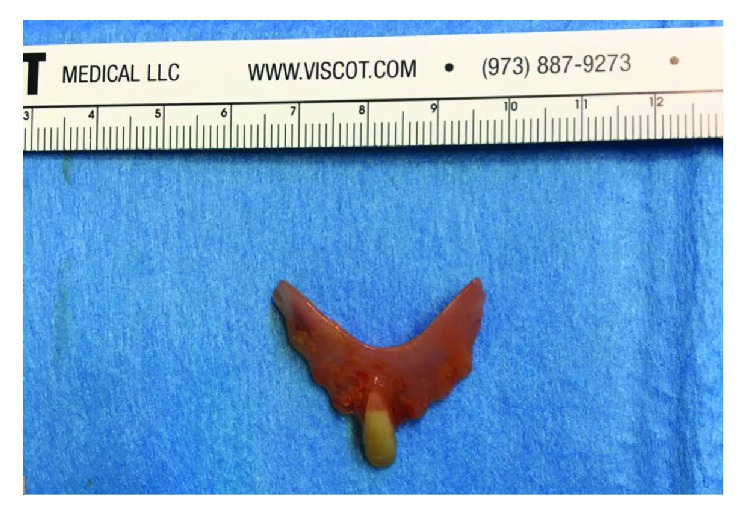
Removable partial lower incisor denture measuring 3 centimeters.

In the operating room, a Weerda scope was advanced orally to the level of the CP muscle. We were not able to visualize a clear CP bridge; however, a large cavity was seen at the level of the muscle with a free edge of a FB seen within. A zero-degree telescope was used to enhance visualization. The distal part of the FB was embedded in the esophageal wall. After gentle blunt dissection, it was successfully removed using long forceps (Figure 3) and was a removable partial lower incisor denture (Figure 4). Inspection of the esophagus after retrieval revealed minimal injury to the esophageal wall and complete resolution of the pouch.

Postoperatively, the patient was well, and her symptoms abated. She was kept under observation for the first 24 hours where her vital signs remained stable. Her diet progressed from soft to regular on the second day, after which she was discharged.

## 3. Discussion

Accidental ingestion of foreign bodies is a problem often faced in the emergency department with acute symptoms or, less frequently, in clinics with insidious presentations. Accidental ingestion and impaction of dental prostheses have been previously reported in literature. The incidence of esophageal denture impaction among FB ingestions ranged between 0.4% and up to 17.6%, and the most common site of impaction was the esophagus [2]. Certain high-risk groups have been mentioned in the literature including patients with confused mental status, alcohol inebriation, general anesthesia, medication overdoses, and neurological diseases such as seizure disorder [3, 4]. However, a systematic review by Kent et al. evaluated published reports of denture swallowing or aspiration over 15 years and concluded that 56% of patients did not have any predisposing factors. They also noted that most retrieved dentures were damaged or loose [5].

Patients with inadvertent denture swallowing may present with a clear history of the ingestion incident with or without additional complaints. They may also have no recollection of the accident and present instead with other vague symptoms that can resemble Zenker diverticulum. The most common symptoms upon presentation are throat discomfort, dysphagia, odynophagia, and persistent FB sensation [1, 6, 7]. The most reliable clinical signs indicative of FB entrapment in the cervical esophagus were neck tenderness and pooling of saliva in the pyriform sinuses [3, 5, 7, 8]. Our patient had no positive signs on clinical examination or flexible fiberoptic laryngoscopy.

The majority of case reports and retrospective studies documenting unintentional denture swallowing reported acute ingestions with patients often presenting within the span of 48 hours [6–8]. However, there were case reports with prolonged denture impactions varying in duration from days to years [3, 4]. Longer durations of denture entrapment have been linked with increased frequency of various complications stressing the importance of early intervention [1, 3]. Some late complications are life threatening such as fistula formation with major vessel erosion, major bronchus fistulas, abscess formation, or bowel perforation. Other possible complications include acute laryngeal edema and stricture or diverticular development [4, 7]. Fortunately, our patient only had a relatively minor complication of pulsion diverticulum formation after a lengthy 2-year course of impaction that resolved immediately after FB extraction.

There are several classifications for pharyngeal/esophageal diverticula.

One of the commonly encountered types of diverticula is Zenker diverticulum, which is an acquired false pulsion diverticulum located just adjacent to the CP muscle that forms because of increased pressure in the pharynx during deglutition leading to herniated mucosa and submucosa through a muscular wall defect. In contrast, our patient had a true pulsion type diverticulum just below the CP muscle bearing a higher resemblance to a Killian Jamieson diverticulum.

The majority of dentures are created from a synthetic resin acrylic termed polymethyl methacrylate. This material is radiolucent, making the diagnosis of impacted dentures more problematic [9]. They may or may not possess a metal attachment/clasp, and delays in discovery occur more often with unclasped dentures. Despite numerous reports of this problem, a radio-opaque denture material alternative has yet to be commercially available possibly due to their increased vulnerability to crack or break [9]. With that in mind, we can logically deduce that lateral neck radiographs would be rarely helpful. If they are performed, however, there are possible signs reported which include air in the esophagus, FB shadow, and salivary pooling with fluid level, in addition to losing the cervical lordosis [10]. Water-soluble contrast studies of the upper gastrointestinal tract would be ideal to detect the location of impaction and presence of a diverticulum and to diagnose other possible causes of dysphagia such as strictures. The utilization of barium studies in cases of suspected FB ingestion has been less favored in the literature as they can make the following endoscopy more challenging by coating the FB. CT imaging offers some advantages over other radiological techniques by its superiority in detecting acrylic based dentures and potential complications [8].

Initially, our patient's management plan included endoscopic diverticulotomy for a Zenker diverticulum; however, it was aborted upon discovery and removal of the culprit FB. There are several approaches for the removal of impacted dental prostheses: observation, endoscopic or open surgical retrieval. Although there had been reports of cases where dentures passed spontaneously, there is limited value for a watchful waiting approach in cases of denture impaction due to their high complication rates [7]. Our present case was managed successfully with endoscopic extraction of the ingested dental prosthesis. Ultimately, the method of removing the FB largely depends on clinical judgment.

## 4. Conclusion

When managing certain patients with dysphagia and odynophagia, a high index of suspicion for FB ingestion is needed even with prolonged symptoms. Emphasis on enquiring about dentures is particularly of value in the elderly, and thorough examination of the oral cavity and dentition is essential. Even though most dentures are radiolucent, imaging studies could still aid in the diagnosis, particularly studies of the upper gastrointestinal tract and CT radiography. Lateral neck radiographs might be useful in detecting radiopaque clasps on dental prostheses. When managing a suspected CP level diverticulum, including Zenker diverticulum, the authors recommend first evaluating the upper esophagus endoscopically, even when planning an open approach, to rule out any concealed FBs.

## Data Availability

Data sharing is not applicable to this article as no datasets were generated or analyzed during the current study.

## Abbreviations

FB: Foreign body
CT: Computed tomography
CP: Cricopharyngeus.
